# SENP3 and USP7 regulate Polycomb-rixosome interactions and silencing functions

**DOI:** 10.1016/j.celrep.2023.112339

**Published:** 2023-04-03

**Authors:** Haining Zhou, Wenzhi Feng, Juntao Yu, Tiasha A. Shafiq, Joao A. Paulo, Jiuchun Zhang, Zhenhua Luo, Steven P. Gygi, Danesh Moazed

**Affiliations:** 1 Howard Hughes Medical Institute, Department of Cell Biology, Blavatnik Institute, Harvard Medical School, Boston, MA, USA; 2 National Laboratory of Biomacromolecules, CAS Center for Excellence in Biomacromolecules, Institute of Biophysics, Chinese Academy of Sciences, Beijing 100101, China; 3 Department of Cell Biology, Blavatnik Institute, Harvard Medical School, Boston, MA, USA; 4 Initiative for Genome Editing and Neurodegeneration, Department of Cell Biology, Blavatnik Institute, Harvard Medical School, Boston, MA, USA; 5 Precision Medicine Institute, the First Affiliated Hospital, Sun Yat-sen University, Guangzhou, China; 6 Lead contact

## Abstract

The rixosome and PRC1 silencing complexes are associated with deSUMOylating and deubiquitinating enzymes, SENP3 and USP7, respectively. How deSUMOylation and deubiquitylation contribute to rixosome- and Polycomb-mediated silencing is not fully understood. Here, we show that the enzymatic activities of SENP3 and USP7 are required for silencing of Polycomb target genes. SENP3 deSUMOylates several rixosome subunits, and this activity is required for association of the rixosome with PRC1. USP7 associates with canonical PRC1 (cPRC1) and deubiquitinates the chromodomain subunits CBX2 and CBX4, and inhibition of USP activity results in disassembly of cPRC1. Finally, both SENP3 and USP7 are required for Polycomb- and rixosome-dependent silencing at an ectopic reporter locus. These findings demonstrate that SUMOylation and ubiquitination regulate the assembly and activities of the rixosome and Polycomb complexes and raise the possibility that these modifications provide regulatory mechanisms that may be utilized during development or in response to environmental challenges.

## In brief

Zhou et al. find that association of the rixosome, which degrades RNA, and Polycomb silencing complexes is regulated by enzymatic removal of ubiquitin and the ubiquitin-like moiety SUMO. The deSUMOylating and deubiquitinating enzymes are associated with the rixosome and Polycomb complexes, respectively, and are required for their silencing functions.

## Graphical Abstract

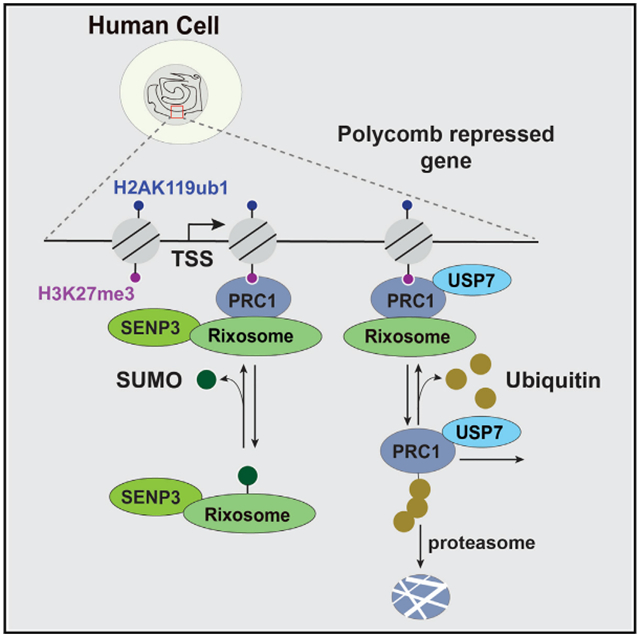

## INTRODUCTION

The Polycomb group (PcG) genes play critical roles in maintenance of stem cell identity and self-renewal and cell lineage commitment, and their loss is associated with developmental abnormalities and cancer.^1-3^ PcG proteins form two major chromatin-modifying complexes, Polycomb repressive complex 1 (PRC1) and Polycomb repressive complex 2 (PRC2).^1,4^ The PRC2 complex catalyzes histone H3K27 trimethylation (H3K27me3) via its EZH2 subunit and binds to nucleosomes containing this modification via its EED subunit.^5-9^ Six different PRC1 complexes have been defined based on the identity of their PCGF subunits.^1^ Canonical PRC1 complexes contain a RING finger E3 ubiquitin ligase (RING1A or B), which catalyzes H2AK119 mono-ubiquitination (H2AK119ub1), a chromobox subunit (CBX2, CBX4, CBX6, CBX7, or CBX8), PHC1, 2, or 3, and PCGF 2 or 4 (PRC1.2 and 1.4).^10-12^ In addition, variant PRC1 (vPRC1) complexes have been identified that lack a CBX subunit and instead contain RINB1A/B associated with RYBP/YAF2 and PCGF1, 3, 5, or 6 (PRC1.1, 1.3, 1.5, and 1.6).^13^ Recent studies suggest that the recruitment of vPRC1 initiates H2K119ub1 that is recognized by PRC2 accessory subunits leading to H3K27 trimethylation.^14-16^ The CBX subunits of cPRC1 complexes then bind to H3K27me3 and mediate silencing via chromatin compaction and other mechanisms.^17-19^

**Figure 1.**
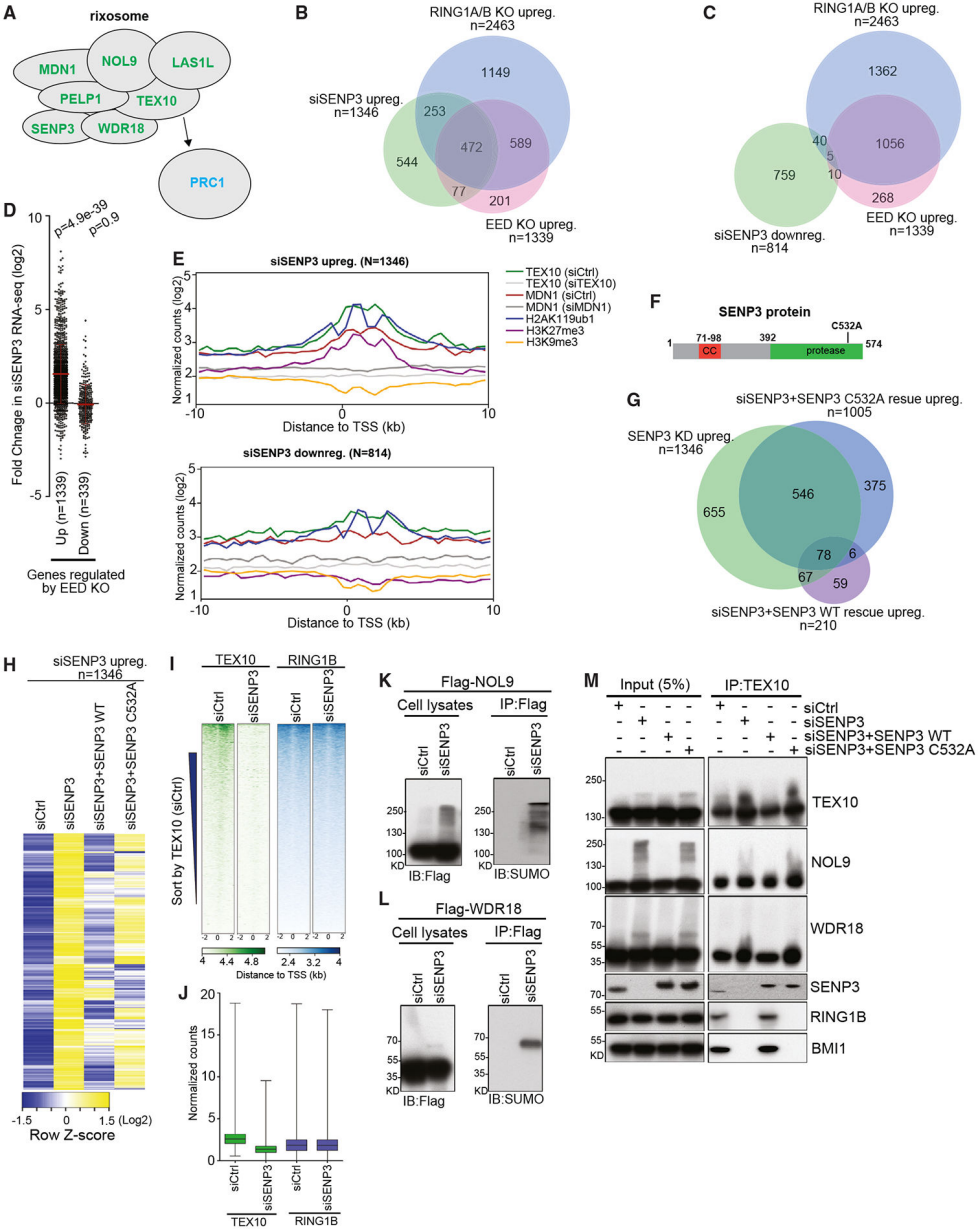
SENP3 is required for Polycomb target gene repression (A) Diagram showing the composition of rixosome. Arrow highlights the interaction of the TEX10 subunit of the rixosome with PRC1. (B and C) Venn diagrams showing overlap among upregulated (B) and downregulated (C) genes in *SENP3* KD with upregulated genes in *EED* KO and *RING1A/B* DKO cells in RNA-seq experiments. Hypergeometric probability p values: siSENP3 upreg vs. *RING1A/B* DKO upreg, 4 x 10^−374^; siSENP3 upreg vs. *EED* KO upreg, 2 x 10^−354^, siSENP3 downreg vs. *RING1A/B* DKO upreg, 1.5 x 10^−7^; siSENP3 downreg vs. *EED* KO, 1.8 x 10^−8^ upreg. (D) Dot plots showing RNA-seq changes of siSENP3 compared with siControl (siCtrl) in the *EED* KO-upregulated and -downregulated sets of genes in RNA-seq experiments with two biological replicates in HEK293FT cells. Data are presented as mean values +/− SEM. p value is from the two-tailed Wilcoxon test. (E) Average distribution of the indicated ChIP-seq reads (log2) for genes upregulated (up) and downregulated genes (down) in siSENP3 RNA-seq experiments from HEK293FT cells. Enrichment levels were normalized with reads per genome coverage. Read counts per gene were summed in 50-nt bins. (F) Diagram of the SENP3 protein and the catalytic point mutation C532A. CC, coiled-coil domains; protease, deSUMOylation catalytic domain. (G) Venn diagrams showing overlap among upregulated genes in SENP3 KD with upregulated genes in SENP3 KD with SENP3 wild-type (WT) or C532A mutant rescue in RNA-seq experiments. (H) Heatmap representations of RNA-seq (two biological replicates) relative gene expression in siControl (siCtrl), siSENP3, siSENP3+SENP3 WT, or siSENP3+SENP3 C532A mutant rescue cells. (I) Heatmap representations of ChIP-seq (two biological replicates) of TEX10 and RING1B in siControl (siCtrl) or siSENP3 cells. Rank order is from most to least TEX10 (siCtrl) signal. Enrichment levels (log2) were normalized with reads per genome coverage. Read counts per gene were averaged in 50-nt bins. (J) Boxplots of normalized enrichment profile of ChIP-seq (two biological replicates) from (I) in TEX10 occupied regions (n = 7,647, where n represents the number of occupied regions). Enrichment levels (log2) were normalized with reads per genome coverage. p = 1.0 x 10^−100^ for TEX10; p = 0.9 for RING1B. p values are from two-tailed Mann-Whitney test. (M) Immunoprecipitations (IPs) showing the association of rixosome subunits TEX10, NOL9, WDR18, and SENP3 and PRC1 subunits RING1B and BMI1 in HEK293FT cells treated with siCtrl, siSENP3, siSENP3+wild-type SENP3 rescue, or siSENP3+SENP3 C532A mutant rescue. Note that in the rescue experiments, SENP3 is RGS-tagged and migrates slower.

The rixosome is a highly conserved complex with essential roles in rRNA processing and ribosome biogenesis.^20^ In addition to its essential functions, in the fission yeast *S. pombe*, the rixosome localizes to heterochromatin and is required for heterochromatin spreading and epigenetic inheritance of a newly established domain of heterochromatin during cell division.^21,22^ We recently showed that the human rixosome associates with PRC1 and localizes to Polycomb target genes.^23^ The rixosome contains seven subunits (Las1L, NOL9, TEX10, WDR18, PELP1, MDN1, and SENP3) and has multiple catalytic activities (Figure 1A).^20^ The TEX10 subunit of the rixosome binds to the RING1B subunit of PRC1 and mediates rixosome chromatin targeting where its RNA degradation activities are required for full silencing of Polycomb target genes.^23^ The SENP3 subunit is a deSUMOylating enzyme whose functional role(s) is not understood.

The ubiquitin-specific protease (USP7) has been shown to interact with both cPRC1 and vPRC1 complexes. USP7 interacts with cPRC1 complexes via the SMCL2 subunit of cPRC1 and was recently shown to also associate with the vPRC1 where it counteracts the TRIM27 E3 ubiquitin ligase.^24,25^ USP7 deubiquitinates several PRC1 subunits, and the inhibition of its activity results in destabilization of PRC1s and impaired silencing of Polycomb target genes.^26^ However, Maat et al. recently reported that purifications of USP7 from the human K562 hematopoietic cell line contained high levels of vPRC1.1 subunits, as well as SMCL2, but very little cPRC1-specific CBX8.^27^ In addition, they found little or no USP7 in immunoprecipitations of tagged cPRC1-specific subunits PCGF2-GFP, PCGF4-GFP, and GFP-CBX2. Based on these observations, it has been suggested that USP7 interacts predominantly with vPRC1.1, and its interaction with SCML2 may occur outside of cPRC1 complexes. However, it remains possible that USP7-cPRC1 interactions are cell type regulated, and it also cannot be ruled out that the specific tags used in the above study may have affected protein-protein interactions.

Here, we show that the association of rixosome and PRC1 complexes is regulated by the SENP3 subunit of the rixosome, which deSUMOylates several rixosome subunits. In addition, our analysis of endogenous cPRC1 complexes and their associated proteins provides strong support for the interaction of USP7 with cPRC1 and USP7-mediated PRC1 deubiquitination. Together with previous findings, our results suggest that the stability of PRC1 complexes and their association with the rixosome are regulated by deubiquitination and deSUMOylation, respectively.

## RESULTS

### The enzymatic activity of SENP3 is required for silencing of Polycomb target genes

To investigate the possible role of SENP3 in silencing, we knocked down SENP3 using siRNA and performed RNA-seq. Knockdown of SENP3 (siSENP3) upregulated a set of genes that largely overlapped with those upregulated in *RING1A/B* double knockout (DKO) and *EED* knockout (KO) in HEK293FT cells (Figure 1B). By contrast, siSNEP3-downregulated genes showed very few overlaps with genes that were upregulated in *RING1A/B* DKO and *EED* KO cells (Figure 1C). Consistently, the genes that were upregulated, but not the genes that were downregulated, in *EED* KO cells were also upregulated in siSENP3 cells (Figure 1D). Furthermore, metagene analysis of chromatin immunoprecipitation sequencing (ChIP-seq) data indicated that relative to siSENP3-downregulated genes, the siSENP3-upregulated ones were more highly enriched for rixosome subunits and Polycomb-catalyzed histone modifications H2K119ub1 and H3K27me3 but not for H3K9me3 (Figure 1E). We obtained similar results using a more stringent cutoff to define changes in gene expression (fold change >5, Figures S1A and S1B). The substitution of cysteine 532 with alanine in SENP3 (C532A) has been shown to disrupt its deSUMOylation activity (Figure 1F).^28^ To investigate further whether SENP3 deSUMOylation activity is required for its silencing functions, we expressed either wild-type SENP3 or SENP3-C532A mutant proteins in siSENP3 cells. Analysis of RNA-seq data showed that wild-type SENP3 but not SENP3-C532A mutant rescued the upregulation of target genes in siSENP3 cells (Figures 1G and 1H). Moreover, siSENP3-upregulated but not siSENP3-downregulated genes overlapped with genes that were upregulated in RING1B-2A mutant cells, in which the interaction of the rixosome with the PRC1 complex is abolished (Figures S1C and S1D). Notably, the overexpression of catalytically dead SENP3 rescued some but not all of the genes that were upregulated in siSENP3 cells, suggesting that SENP3 had both catalytic and non-catalytic functions (Figures 1G and 1H). Together, these results indicated that the deSUMOylation activity of SENP3 was required for most of its silencing functions.

### SENP3 activity is required for chromatin localization of the rixosome and its association with PRC1

Because the rixosome localizes to Polycomb target genes,^23^ we tested the requirement for SENP3 in genome-wide chromatin localization of the rixosome. As shown by heatmaps in Figure 1I and quantification in Figure 1J, the localization of the TEX10 subunit of the rixosome to promoter regions (+/−2 kb from transcription start site [TSS]) was reduced in SENP3 knockdown cells, suggesting SENP3 was required for association of the rixosome with chromatin. By contrast, the localization of the RING1B subunit of PRC1 was unaffected in SENP3 knockdown cells (Figures 1I and 1J). By western blotting of cell lysates and immunoprecipitations, we observed slower migrating forms of Flag-tagged rixosome subunits NOL9 and WDR18 in siSENP3 cells (Figures 1K and 1L). Furthermore, using antibodies that recognize native proteins, we observed slower migrating forms of TEX10, NOL9, and WDR18, and the change in their migration was rescued by wild-type but not catalytically dead SENP3-C532A, suggesting that the slower migrating forms resulted from a defect in SENP3-mediated deSUMOylation (Figure 1M, left panels). TEX10 immunoprecipitations showed that neither SENP3 nor its catalytic activity were required for the integrity of the rixosome (Figure 1M). In striking contrast, the association of PRC1 subunits RING1B and BMI1 (PCGF4) with TEX10/rixosome was lost in siSENP3 cells, and this loss was rescued by the expression of wild-type but not catalytically dead SENP3-C532A protein, indicating that SENP3-catalyzed deSUMOylation was required for the association of rixosome with PRC1 (Figure 1M). Thus, the requirement for deSUMOylation activity of SENP3 in silencing may be explained by its role in promoting the association of rixosome with PRC1.

### SENP3 is required for silencing of Polycomb target genes in human ESCs

We next tested the function of SENP3 in gene regulation in another cell line, human embryonic stem cells (hESCs). Like HEK293FT cells, RNA-seq experiments showed that siSENP3-upregulated genes largely overlapped with si*RING1B* and *EZH1/2* DKO-upregulated genes, while siSNEP3-downregulated genes showed very few overlaps with the *RING1B* KD and *EZH1/2* DKO-upregulated genes (Figures S2A-S2D). Metagene analysis of ChIP-seq data indicated that the siSENP3-, siRING1B-, and *EZH1/2* DKO-upregulated but not siSENP3-downregulated genes were enriched for Polycomb-catalyzed histone modifications H2AK119ub1 and H3K27me3 but not for H3K9me3 (Figures S2E-S2H). Therefore, the requirement for SENP3 in repression of Polycomb target genes is not cell type specific.

### USP7 is associated with canonical PRC1 and is required for silencing of Polycomb target genes

**Figure 2.**
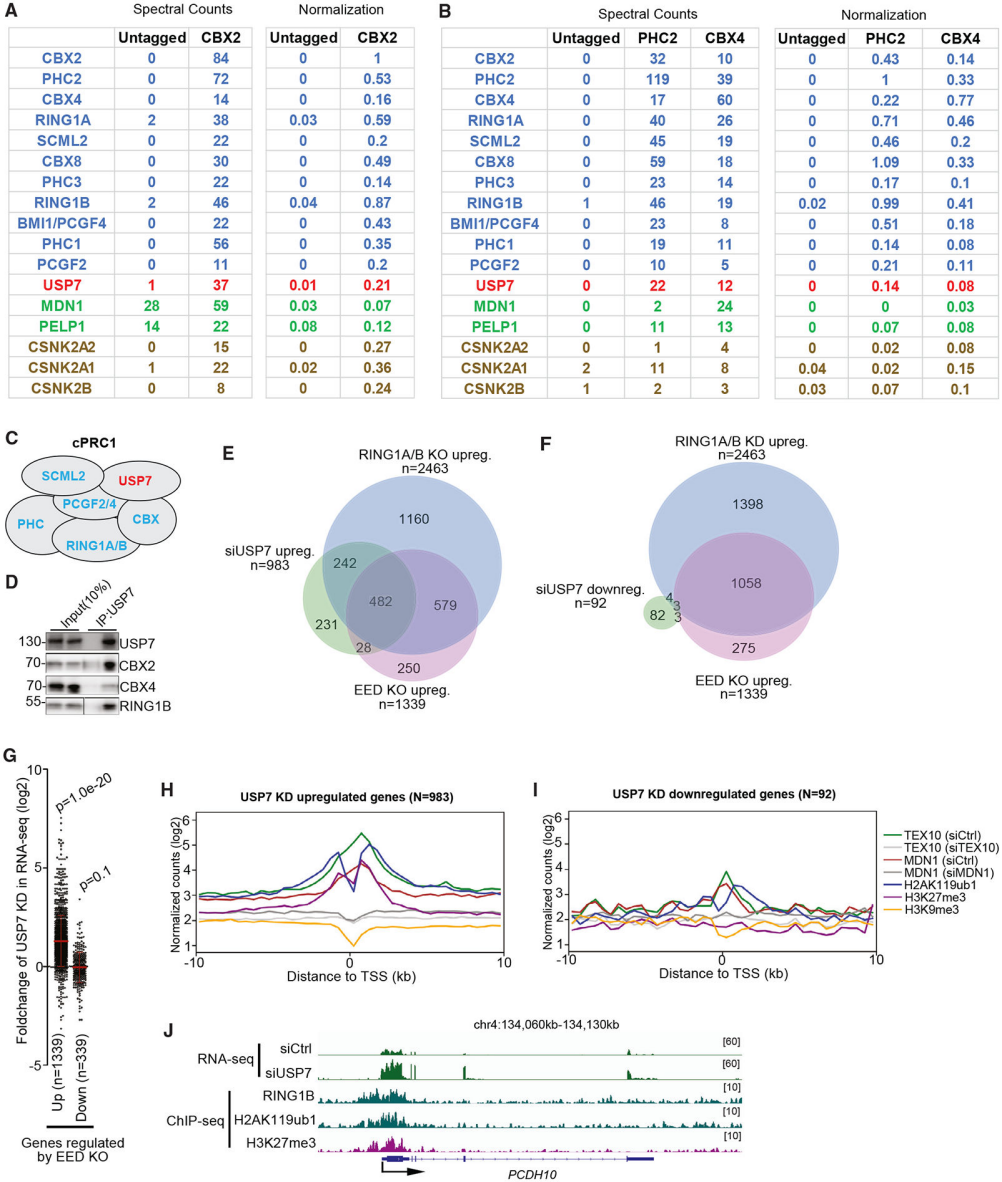
USP7 associates with cPRC1 and is required for Polycomb target gene repression (A) Table showing spectral counts of IP (from two biological replicates) samples from untagged and CBX2 endogenously Flag-tagged HEK293FT cells. Normalized spectral counts on the right side were obtained by dividing total spectral counts by the number of amino acids (length) of each protein. The normalized spectral counts for the bait protein (CBX2, CBX4, or PCH2) were set to 1, and the counts for co-purifying proteins are presented relative to the bait. (B) Table showing spectral counts of IP (from two biological replicates) samples from untagged and PHC2 or CBX4 endogenously Flag-tagged HEK293FT cells. (C) Schematic diagram showing the composition of canonical PRC1 complex (cPRC1). In HEK293FT cells, cPRC1 contains SCML2 and USP7 in addition to its core components RING1A/B, PCGF2/4, PHC1/2/3, and CBX2/4/6/7/8 (only CBX2, 4, and 8 are expressed in HEK293FT). (D) Immunoprecipitation (IP) experiments showing the association of USP7 with cPRC1 subunits CBX2, CBX4, and RING1B in HEK293FT cells. (E and F) Venn diagrams showing overlap among upregulated (E) and downregulated (F) genes in siUSP7 with upregulated genes in *EED* KO and *RING1A/B* DKO cells in RNA-seq experiments. Hypergeometric probability p values: siUSP7 upreg vs. *RING1A/B* DKO upreg, 2.8 x 10^−517^; siUSP7 upreg vs. *EED* KO, 9.6 x 10^−393^ upreg, siUSP7 downreg vs. *RING1A/B* DKO upreg, 0.2; siSENP3 downreg vs. *EED* KO upreg, 0.4. (G) Dot plots showing RNA-seq changes (from two biological replicates) of siUSP7 compared with siControl (siCtrl) in the *EED* KO-upregulated and -downregulated sets of genes in RNA-seq experiments in HEK293FT cells. Data are presented as mean values +/− SEM. p value is from the two-tailed Wilcoxon test. (H and I) Average distribution of the indicated ChIP-seq reads (log2) (from two biological replicates) for genes upregulated (H) and downregulated genes (I) in siUSP7 RNA-seq experiments from HEK293FT cells. Enrichment levels were normalized with reads per genome coverage. Read counts per gene were summed in 50-nt bins. (J) Genome browser snapshots of RNA-seq experiments showing expression levels of the indicated genes in siCtrl and siUSP7 cells and ChIP-seq showing enrichment of RING1B, H2AK119ub1, and H3K27me3. Enrichment levels were normalized with reads per genome coverage.

We analyzed our previous purifications of cPRC1-specific subunits PHC2 and CBX4.^23^ In addition to rixosome subunits MDN1 and PELP1, high levels of USP7 and CK2 subunits (CSNK2A1, CSNK2A2, and CSNK2B) were copurified with each PRC1 subunit, PHC2 and CBX4^23^(Figures 2A-2C). To further determine whether USP7 associates with cPRC1 complexes, we performed IP-mass spectrometry experiments using endogenously Flag-tagged CBX2, another cPRC1-specific subunit. The results showed that CBX2 purifications were enriched for all core cPRC1 subunits: PHC2, RING1A, RING1B, BMI1/PCGF4, PCGF2, and SCML2 (Figure 2A). Furthermore, CBX4 and CBX8 were enriched in the CBX2-Flag immunoprecipitations at similar levels to the core cPRC1 subunits such as RING1A, RING1B, PHC1, and PHC3 (Figure 2A), likely due to the previously reported multimerization of PRC1 complexes.^29,30^ Normalization of spectral counts for tryptic peptides identified in each purification, which correlates with protein abundance, indicated that each of the above proteins were present in CBX2-Flag immunoprecipitations at similar levels (Figure 2A). Consistent with these results, CBX4 immunoprecipitations were enriched for all core cPRC1 subunits and for CBX2 and CBX8 (Figure 2B). In addition to core cPRC1 subunits, other CBX proteins, and rixosome subunits, CBX2 and CBX4 purifications were enriched for USP7 and CSNK2 proteins (Figures 2A and 2B). We further used immunoprecipitation with an antibody that recognizes USP7 to show that both cPRC1-specific (CBX2 and CBX4) and a cPRC1- and vPRC1-common protein were associated with USP7 (Figure 2D). Together, these results indicate that USP7 interacts with cPRC1 complexes.

We next performed siRNA knockdown and RNA-seq experiments in HEK293FT cells to test whether USP7 was required for silencing of Polycomb target genes. Consistent with previous results in K562 cells,^27^ analysis of the RNA-seq data showed that siUSP7-upregulated genes largely overlapped with *RING1A/B* DKO- and *EED* KO-upregulated genes (Figure 2E). By contrast, siUSP7-downregulated genes showed very few overlaps with *RING1A/B* DKO- and *EED* KO-upregulated genes (Figure 2F). We obtained similar results using a more stringent cutoff to define changes in gene expression (fold change >5, Figures S3A and S3B). Consistently, dot plot analysis showed that the upregulated but not the downregulated genes in *EED* KO cells were preferentially upregulated in siUSP7 cells (Figure 2G). Furthermore, metagene analysis of ChIP-seq data indicated that the siUSP7 upregulated but not downregulated genes were enriched for rixosome subunits and Polycomb-catalyzed histone modifications but not H3K9me3 (Figures 2H and 2I). As an example, at the single-gene level, *PCDH10* gene is upregulated in siUSP7 cells (Figure 2J).

To investigate whether the deubiquitinating activity of USP7 was required for Polycomb-mediated gene silencing, we preformed RNA-seq in HEK293FT cells treated with a selective USP7 inhibitor FT671, a newly described and highly specific inhibitor.^31^ RNA-seq experiments showed that genes upregulated by USP7 inhibition largely overlapped with *RING1A/B* DKO- and *EED* KO-upregulated genes (Figures S3C-S3F). In contrast, the genes downregulated by USP7 inhibition showed very few overlaps with *RING1A/B* DKO- and *EED* KO-upregulated genes (Figures S3C-S3F). Consistently, dot plot analysis showed that the upregulated but not the downregulated genes in *EED* KO cells were preferentially upregulated by inhibition of USP7 (Figure S3G). These results provide an independent validation of the siUSP7 data and in addition suggest that the deubiquitinating activity of USP7 is required for silencing of Polycomb target genes.

### USP7 activity is required for integrity of PRC1

**Figure 3.**
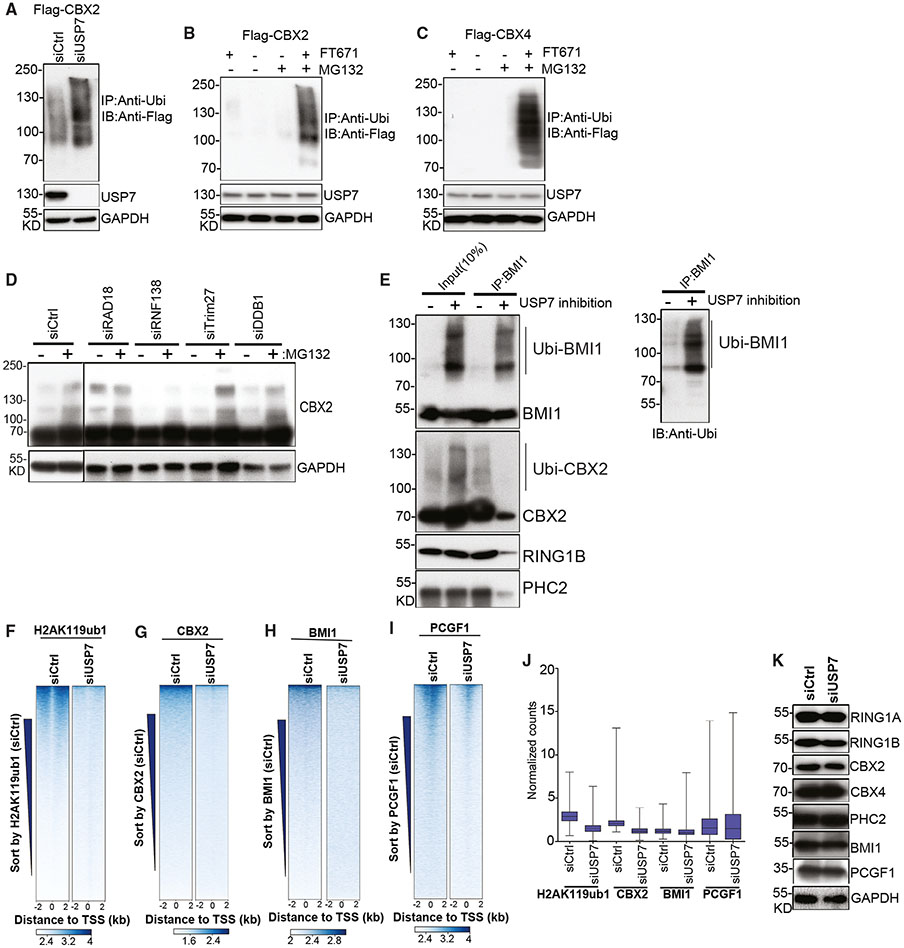
USP7 deubiquitylates cPRC1 subunits and promotes cPRC1 binding to chromatin (A) Immunoblotting showing anti-ubiquitin immunoprecipitations (IPs) in Flag-CBX2 HEK293FT cells treated with siCtrl or siUSP7 (3 days) and 5 μM MG132 for 1 day. GAPDH served as input control. (B) Immunoblotting showing anti-ubiquitin immunoprecipitations (IPs) in Flag-CBX2 HEK293FT cells not treated or treated with 5 μM MG132, 1 μM FT671, or 1 μM FT671 + 5 μM MG132 for 1 day. GAPDH served as input control. (C) Immunoblotting showing anti-ubiquitin immunoprecipitations (IPs) in Flag-CBX4 HEK293FT cells not treated or treated with 5 μM MG132, 1 μM FT671, or 1 μM FT671 + 5 μM MG132 for 1 day. GAPDH served as input control. (D) Immunoblotting showing Flag-CBX2 HEK293FT from cells treated with the indicated siRNA (3 days) and 5 μM MG132 or not treated for 1 day. All cells were treated with 1 μM FT671. GAPDH served as input control. (E) Immunoprecipitation (IP) experiments showing the interactions between PRC1 complex subunits BMI1, CBX2, RING1B, and PHC2 in HEK293FT cells with and without treatment with 1 μM FT671 for 1 day (left) in cells treated with 5 μM MG132. Immunoblotting detection of ubiquitylated form of BMI1 proteins in anti-BMI1 immunoprecipitation samples (right). (F) Heatmap representations of ChIP-seq of H2AK119ub1 in siControl (siCtrl) or siUSP7 cells (from two biological replicates). Rank order is from most to least H2AK119ub1 (siCtrl) signal. (G–I) Heatmap representations of ChIP-seq of CBX2 (G), BMI1 (H), and PCGF1 (I) from siControl (siCtrl) or siUSP7 cells (from two biological replicates). Rank order is from most to least CBX2 (siCtrl) signal. Enrichment levels (log2) were normalized with reads per genome coverage. Read counts per gene were averaged in 50-nt bins for (F)–(I). (J) Boxplots of normalized enrichment profile of ChIP-seq (from two biological replicates) from (F) to (I) in H2AK119ub1 occupied regions (n = 6,820, where n represents occupied regions). Enrichment levels (log2) were normalized with reads per genome coverage. p = 1.0 x 10^−100^ for H2AK119ub1; p = 1.0 x 10^−100^ for CBX2; p = 1.4 x 10^−97^ for BMI1; p = 1.0 x 10^−8^ for PCGF1. p values are from two-tailed Mann-Whitney test. (K) Immunoblotting showing expression levels of indicated proteins in HEK293FT cells treated with siCtrl or siUSP7 (3 days). GAPDH served as loading control.

Using four different USP7 inhibitors that show different efficacies,^31-35^ we examined the effects of USP7 inhibition on PRC1 subunit gel migration by immunoblotting. We observed slower migrating forms of CBX2, CBX4, and BMI1 in western blots of extracts from P5091-treated cells, suggesting that these PRC1 subunits may be targets of USP7-mediated deubiquitylation (Figures S4A-S4M). To investigate whether the slower migrating species were indeed ubiquitinated species, we performed anti-ubiquitin IP from Flag-CBX2 cells treated with the MG132 proteasome inhibitor with and without siRNA-mediated USP7 knockdown. The results showed that ubiquitin immunoprecipitations contained Flag-CBX2, suggesting that CBX2 was ubiquitinated and targeted for proteasomal degradation (Figure 3A). Furthermore, the intensity of the Flag-CBX2 signal was stronger in siUSP7 relative to siControl cells, suggesting that USP7 deubiquitinated CBX2 (Figure 3A). USP7-dependent CBX2 deubiquitylation was further demonstrated by a striking increase in the levels of ubiquitinated Flag-CBX2 in ubiquitin immunoprecipitations from cells treated with the USP7 inhibitor FT671 (Figure 3B). Similar experiments showed that CBX4 was also ubiquitinated in MG132+FT671 treated cells (Figure 3C). These results identify the CBX2 and CBX4 subunits of cPRC1 as ubiquitinated proteins that are targeted by USP7.

To identify the ubiquitin ligase responsible for CBX2 and CBX4 ubiquitination, we focused on those ligases that are enriched in immunoprecipitations in Flag-CBX2 compared with untagged cells. We performed siRNA knockdown of each ligase in cells that were treated with the FT671 USP7 inhibitor. Only RNF138 KD exhibited a significant reduction in ubiquitination of Flag-CBX2 and CBX4 (Figures 3D and S4N-S4R), suggesting that RNF138 ubiquitinates the CBX2 and CBX4 proteins.

To test whether USP7 activity affected the integrity of PRC1 complex, we carried out immunoprecipitation experiments. We observed a striking reduction in enrichment of PRC1 subunits RING1B, CBX2, BMI1, and PHC2 in BMI1 (PCGF4) immunoprecipitations in the presence of the USP7 inhibitor FT671, suggesting that USP7 activity was required for PRC1 integrity (Figure 3E). Consistent with these results, the co-sedimentation of CBX2 and BMI1 in sucrose gradients was greatly reduced in extracts from cells treated with the FT671 USP7 inhibitor compared with untreated cells (Figure S4S). Furthermore, knockdown of USP7 resulted in reduced enrichment of H2AK119ub, CBX2, BMI1, and PCGF1 at promoter regions in ChIP-seq experiments (Figures 3F-3J). The levels of RING1A, RING1B, CBX2, CBX4, PCH2, BMI1, and PCGF1 proteins were unaffected in siUSP7 cells (Figure 3K). These results suggest that USP7-mediated deubiquitylation stabilizes the PRC1 complex assembly and regulates its association with its target loci on chromatin.

### SENP3 and USP7 are required for silencing at an ectopic locus

**Figure 4.**
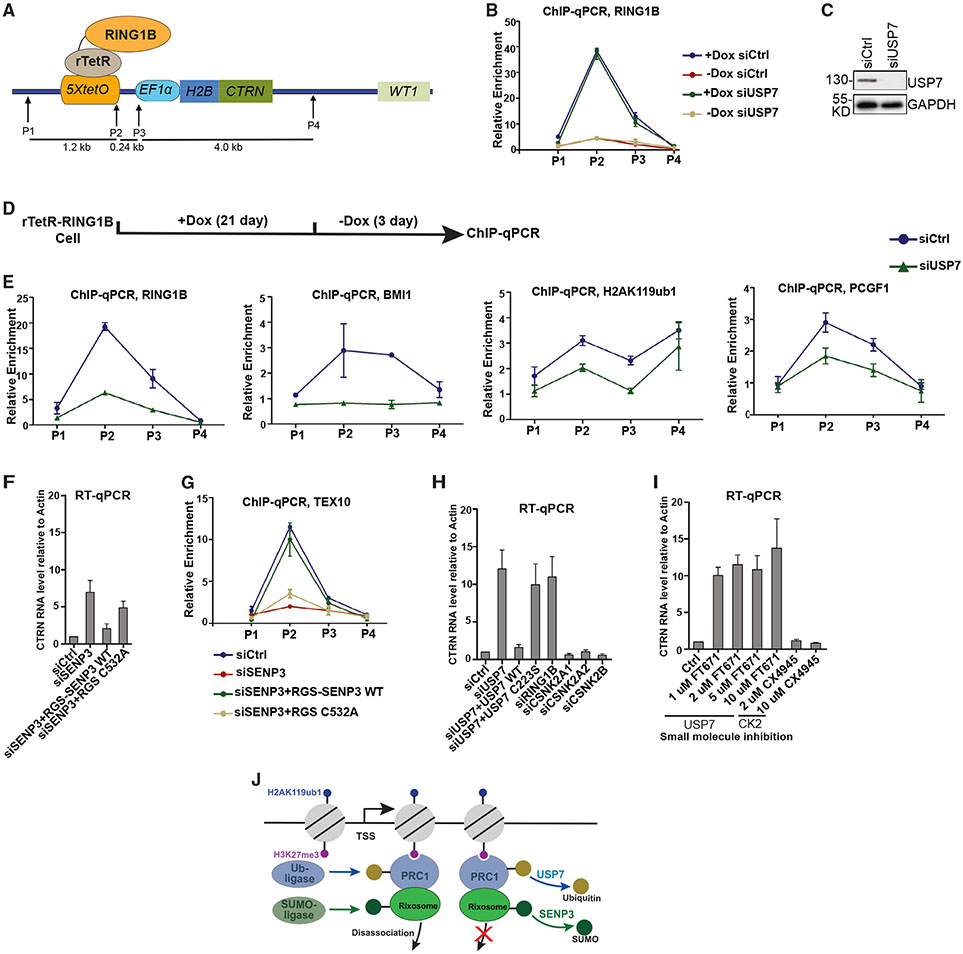
SENP3 and USP7 are required for ectopic Polycomb target gene silencing (A) Diagram for the construction of cell lines with *5xtetO-H2B-CITRINE* (*H2B-CTRN*) reporter gene expressing rTetR-RING1B fusion proteins. (B) ChIP-qPCR analysis of RING1B localization with or without 21-day doxycycline treatment in both siCtrl- and siUSP7-treated H2B-CTRN reporter cells. Upon release from doxycycline (−Dox) for 3 days cells were treated with siCtrl or siUSP7. ChIP signals were normalized to *GAPDH*. Dots represent two biological replicates. Data are presented as mean values +/− SEM. (C) Immunoblots showing USP7 protein expression levels in HEK293FT cells treated with siCtrl or siUSP7 (3 days). GAPDH served as loading control. (D) Diagram for the experimental design with H2B-CTRN reporter cells. Silencing was established by growth in doxycycline-containing medium (+Dox) for 21 days. Cells were then grown in −Dox medium for 3 days. (E) ChIP-qPCR analysis of the indicated proteins and H2AK119ub1 in H2B-CTRN reporter cell lines 3 days after growth in −Dox medium. Upon release from doxycycline, cells were treated with siCtrl or siUSP7. ChIP signals were normalized to *GAPDH*. Dots represent two biological replicates. Data are presented as mean values +/− SEM. (F) qRT-PCR analysis of RNA levels of H2B-CTRN in the indicated siRNA-treated and SENP3-rescued HEK293FT cells 3 days after growth in −Dox medium. RNA expression levels were normalized to *ACTB*, and every knockdown was normalized to siCtrl. Data are presented as mean values +/− SEM from three biological replicates. (G) ChIP-qPCR analysis of TEX10 localization 3 days after growth in −Dox medium in indicated siRNA-treated and SENP3-rescued H2B-CTRN reporter HEK293FT. ChIP signals were normalized to *GAPDH*. Dots represent two biological replicates. Data are presented as mean values +/− SEM. (H) qRT-PCR analysis of RNA levels of H2B-CTRN in the indicated siRNA-treated and USP7-rescued HEK293FT cells 3 days after growth in −Dox medium. siRING1B is presented as a positive control. The effect of knocking down casein-specific kinases is presented on the right. RNA expression levels were normalized to *ACTB*, and every knockdown was normalized to siCtrl. Data are presented as mean values +/− SEM from three biological replicates. (I) qRT-PCR analysis of RNA levels of H2B-CTRN in the indicated small molecules treatment with indicated concentration 3 days after growth in −Dox medium. RNA expression levels were normalized to *ACTB*, and every knockdown was normalized to siCtrl. Data are presented as mean values +/− SEM from three biological replicates. (J) Model for the protective roles of SENP3 and USP7 in rixosome- and PRC1-mediated gene silencing.

To further test the roles of SENP3 and USP7 in Polycomb repression and regulation of PRC1-rixosome binding to chromatin, we fused the RING1B subunit of PRC1 to the bacterial reverse tetracycline repressor (rTetR-RING1B) and expressed it in the cells with a reporter gene at a euchromatic locus (*5xtetO-CTRN*)^23^ (Figure 4A). Doxycycline treatment successfully induced RIGN1B tethering, which was not affected by depletion of USP7 (Figures 4B and 4C). To test whether PRC1 binding to the reporter locus was regulated by USP7, cells were released from growth in doxycycline-containing medium for 3 days, which results in dissociation of the rTetR-RING1B from the reporter locus. ChIP-qPCR experiments showed that both RING1B and BMI1 were recruited to the ectopic locus, and knockdown of USP7 greatly reduced this association (Figures 4D and 4E). Consistently, depletion of USP7 resulted in reduced enrichment of H2AK119ub1 at the reporter locus (Figure 4E). These results further support a direct role for USP7 in regulation of PRC1 recruitment and function.

We next tested the effect of depletion of SENP3 and USP7 and the requirement for their catalytic activities on silencing of the above *5xtetO-CTRN* reporter gene. We performed siRNA knockdown experiments 3 days after the release of rTetR-RING1B (−doxycycline). Under these conditions, depletion of SENP3 and USP7 resulted in strong derepression of the *5xtetO-CTRN* reporter (Figures 4F and 4H).^26,28^ The derepression of *5x-tetO-CTRN* by SENP3 and USP7 depletion was rescued by the expression of wild-type but not catalytic dead SENP3 and USP7 (Figures 4F and 4H). Consistently, depletion of SENP3 resulted in reduced association of the rixosome subunit TEX10 with the report locus, which was rescued by wild-type SENP3 but not catalytically dead SENP3 (Figure 4G). Furthermore, USP7 inhibition by FT671 induced derepression of the reporter gene (Figure 4I). In contrast, the knockdown of CK2 subunits (which were copurified with CBX2 in IP-MS experiments) or inhibition of CK2 kinase activity did not derepress the *CTRN* reporter (Figures 4H and 4I). Therefore, similar to the case for endogenous loci, SENP3 and USP7 contribute to silencing at an ectopic locus.

## DISCUSSION

Our findings demonstrate roles for deSUMOylation and deubiquitination in the regulation of the rixosome and Polycomb complexes, respectively. Polycomb repressed domains can therefore be dynamic and in principle may be regulated by change in SENP3 or USP7 activity. The possible regulation of the SENP3 and USP7 during changes in cell states or in response to environmental signals may provide a mechanism for modulation of silencing at certain genes. Furthermore, the continuous requirement for SENP3 and USP7 suggests that Polycomb domains are vulnerable to modification by SUMO and ubiquitin ligases and must be continuously protected to ensure effective gene repression (Figure 4J).

The SUMO and ubiquitin-proteasome pathways play central roles in the regulation of protein abundance and activity to maintain cellular homeostasis.^36-39^ SENP3-mediated deSUMOylation has been reported to regulate a diverse array of processes including DNA replication and repair, transcriptional activation of hypoxia-induced genes, cytosolic DNA sensing, MLL1/MLL2 methyltransferase complex activity, mitochondrial autophagy, and mitotic spindle assembly.^37,40-47^ Additionally, SUMOylation of the PELP1 subunit of the rixosome and its deSUMOylation by SENP3 were previously shown to regulate the association of the rixosome with pre-60S ribosomal particles and their nucleolar localization.^48,49^ Our findings demonstrate that SENP3-mediated deSUMOylation also regulates the interaction of the rixosome with the PRC1 complex, without affecting the integrity of the rixosome. This observation raises the possibility that rixosome-mediated degradation of RNA in Polycomb domains may be regulated by signaling pathways that act on SENP3. In this regard, SENP3 expression is greatly upregulated during the differentiation of myoblasts to myotubes and is required for deSUMOylation and activation of the SETD7 H3K4 methyltransferase.^42^ SENP3 levels are also modulated in response to oxidative stress, which leads to the stabilization of SENP3 and its redistribution from nucleoli to the nucleoplasm.^41^ SENP3 regulates the association of the HIF-1α transcription factor with the p300 co-activator by deSUMOylating p300 and is required for oxidative-stress-induced transcription activation.^40^ SENP3 therefore regulates both transcriptional repression (this study) and activation processes,^40-42^ possibly in a cell-type- and signaling-dependent manner.

The USP7 ubiquitin protease is a master regulator of numerous genome stability pathways, some of which are also regulated by SENP3.^37,50^ USP7 was previously reported to associate with PRC1 complexes and with the EZH2 subunit of PRC2, but whether it associates with both vRPC1 and cPRC1 had remained unclear.^24-27,51^ Another ubiquitin protease, USP11, has also been reported to interact with PRC1 but was not detected in our cPRC1 immunoprecipitations.^26^ Our findings clearly demonstrate that USP7 associates with the cPRC1 complex and deubiquitinates its CBX2 and CBX4 subunits. USP7-dependent deubiquitination is required for the assembly of an intact cPRC1 complex, raising the possibility that changes in USP7 activity or abundance may regulate Polycomb domains. Interestingly, USP7 has also been reported to function as a SUMO deubiquitinase, which protects SUMOylated proteins from ubiquitination and proteasomal degradation.^52^ In our study, we have not examined the effect of USP7 depletion on the stability of SUMOylated rixosome subunits. However, since PRC1 integrity is compromised in USP7-depleted cells, and because USP7 is associated specifically with PRC1 and not the rixosome, it seems unlikely that it acts as a SUMO deubiquitinase for rixosome subunits.

In *Drosophila*, USP7 deubiquitinates histone H2BK120ub1 and helps to repress the transcription of specific genes.^53^ USP7 may therefore also act through deubiquitination of non-PRC1 substrates to facilitate Polycomb silencing.

Alterations in Polycomb silencing are a hallmark of many cancers.^3,54,55^ The stringent control of Polycomb silencing by SENP3 and USP7 suggests that these enzymatic pathways may be useful targets for anti-cancer drugs. Indeed, USP7 has emerged as a target for drugs that target specific cancers. However, the large number of pathways that are regulated by these enzymes suggests that their inhibition may be undesirable. A greater knowledge of the nature of association of SENP3 and USP7 with the rixosome and PRCs may lead to better strategies for targeted inhibition of their interactions.

### Limitations of the study

Our findings show that deSUMOylation and deubiquitination regulate the association of the PRC1 and rixosome complexes and the stability of cPRC1 complexes but do not address how these modifications are regulated during development or response to environmental changes. Although we find that USP is physically associated with the cPRC1 complex, our findings are consistent with the possibility that USP7 is a dynamic subunit of both cPRC1 and vPRC1 complexes. Our results suggest that ubiquitination inhibits the assembly of cPRC1 subunits into a complex. Consistent with this conclusion, loss of USP7-mediated deubiquitination results in reduced co-immunoprecipitation of the BMI1 and CBX2 subunits of cPRC1, but we note that we also detected a slight interaction between BMI1 and ubiquitinated CBX2 species.

## STAR★METHODS

### RESOURCE AVAILABILITY

#### Lead contact

Further information and requests for resources and reagents should be directed to and will be fulfilled by the lead contact, Danesh Moazed, danesh@hms.harvard.edu.

#### Materials availability

This study did not generate new unique reagents.

#### Data and code availability

- The Mass Spectrometry raw data were deposited with accession number: PXD027966. The raw and processed high-throughput sequencing data have been deposited at NCBI Gene Expression Omnibus under ID code GEO: GSE197030. This paper analyzes existing publicly available data. These accession numbers for the datasets are listed in the key resources table.
- This paper does not report original code.
- Any additional information required to reanalyze the data reported in this paper is available from the lead contact upon request.

The link: https://star-methods.com/?rid=KRT63c14857423ca.

### EXPERIMENTAL MODEL AND SUBJECT DETAILS

#### Cell lines

HEK293FT (ThermoFisher, R70007) cells were cultured in DMEM containing 10% fetal calf serum. Human ES cells (H9, WiCell Institute) were cultured on matrigel coated plates in E8 medium. To generate knockouts in Human ES cells, 0.6 μg sgRNA was incubated with 3 μg SpCas9 protein for 10 minutes at room temperature and electroporated into 2x10^5^ cells. Mutants were identified by Illumina MiSeq and further confirmed by Western blot.

### METHOD DETAILS

#### USP7 inhibition

For USP7 inhibition, 1 μM FT671^31^, 20 μM P5091,^33^ 20 μM P22077,^34^ 20 μM HBX19818,^35^ and 20 μM HBX41108^32^ were used. We initially used P5091, P22077, HBX19818, and HBX41108 as USP7 inhibitors, which were reported earlier (Altun et al., 2011; Chauhan et al., 2012; Colland et al., 2009; Kessler, 2014). A new inhibitor (FT671) was later reported, which seemed to be more efficent and specific on USP7 inhibition (Turnbull et al., 2017). We therefore switched to FT671 and mostly used it for all further experiments on USP7 inhibition.

#### RNAi

For siRNA-mediated knockdown, LipofectamineTM RNAiMAX transfection reagent (Invitrogen) and siRNA (200 nM) were used to transfect the cells by following the manufacturer’s instructions. All the siRNAs were synthesized by Dharmacon and are listed in Table S1.

#### Immunoprecipitation and mass spectrometry analysis

To prepare chromatin-enriched fractions, cells were washed with PBS and then resuspended in ice-cold hypotonic buffer (10 mM HEPES, pH7.9, 1.5 mM MgCl2, 10 Mm KCl, 0.2 mM PMSF, 0.2 mM DTT) and incubated on ice for 10 min. Cell membranes were then disrupted by douncing 10 times. Nuclei were pelleted by centrifugation at 2,000xg for 3 min, resuspended in cell lysis buffer (50 mM HEPES, pH 7.4, 150 mM NaCl, 1 mM MgCl2, 1 mM EGTA, and 0.5% Triton X-100) by pipetting for 3 min, and pelleted by centrifugation at 2,000xg for 10 min to obtain a chromatin fraction. The chromatin pellet was resuspended in IP buffer (20 mM Tris-HCl, pH 7.9, 250 mM NaCl, 10% Glycerol, 2.5 mM MgCl2, 0.5 mM CaCl2, 0.1 mM EDTA, and 0.5% Triton X-100) containing protease inhibitor cocktail (5056489001, Sigma) and 1 mM DNase I. Chromatin was digested for 2 h at 4°C and centrifuged at 10,000xg for 10 min. The supernatant was then incubated with antibodies and immune complexes were collected using Dynabeads Protein A/G (ThermoFisher). For immunoblotting, beads were boiled for 5 min in SDS loading buffer. Mass Spectrometry and data analysis were performed as described previously.^23,56^ Briefly, beads-binding proteins were eluted with 0.5 M NH_4_OH. Proteins were then resuspended in 200 mM EPPS pH 8.5 and digested with 5 ng/ul trypsin. Label-free mass spectrometry data were collected using a Q Exactive mass spectrometer (Thermo Fisher Scientific, San Jose, CA). Mass spectra were processed using a Comet-based in-house software pipeline. MS spectra were converted to mzXML using a modified version of ReAdW.exe.

#### Sucrose gradient sedimentation assays

Sucrose gradient sedimentation assays were performed as described previously with modifications.^23^ Flag-CBX2 and Flag-RING1B were purified from HEK293FT cells that were either untreated (DMSO control) or grown in medium containing 1uM Ft671 for 24 hours using anti-Flag magnetic beads (Sigma, M8823). Bound protein was eluted with 500ng/ul 3×Flag peptides (APExBIO, A6001) in elution buffer (20 mM Hepes-KOH, pH7.5, 100 mM KOAc, 5 mM Mg(OAc)_2_, 1 mM EDTA, 10% Glycerol). Sucrose gradients (10%-30%) were prepared, layered with 50 uL of the eluate from either the Flag-CBX2 or Flag-RING1B purification and centrifuged for 16 h at 4 °C at 35,000 rpm in anTLS-55 rotor. Nine fractions were collected from 1.8 ml gradients by pipetting 200 uL fractions from the top of each gradient. Proteins were then captured using StrataClean resin (Agilent, 400714). Protein samples were boiled in loading buffer (62.5 mM Tris-HCl, pH 6.8, 2% SDS, 10% glycerol, 0.01% bromophenol blue) for 5 min at 96 °C and analyzed by immunoblotting.

#### RT-qPCR

Total RNA was extracted using the RNeasy Plus kit (74134, Qiagen) and reverse transcribed into cDNA using gene-specific primers and reverse transcription kit (18090010, ThermoFisher). cDNA was analyzed by running PCR on a QuantStudio 7 Flex Real Time PCR System (Applied Biosystem). All reactions were performed using 125 ng RNA in a final volume of 10 μl. PCR parameters were 95°C for 2 min and 40 cycles of 95°C for 15s, 60°C for 15s, and 72°C for 15 s, followed by 72°C for 1 min. All the qPCR data presented were at least three biological replicates.

#### RNA-seq

Total RNA was isolated from human cells with an RNA purification kit (74134, Qiagen) and genomic DNA was removed by DNA binding columns in the kit. Two μg of total RNA was used for RNA-seq library construction. Poly(A)-tailed mRNA was isolated by poly(A) selection beads and further reverse transcribed to cDNA. The resulting cDNA was ligated with adapters, amplified by PCR, and further cleaned to obtain the final libraries, which were sequenced on an Illumina Hiseq machine (Novogene) to obtain 150 bp paired-ended reads.

RNA-seq reads were pseudo aligned using Kallisto 0.45.1. An index was generating using the Ensembl hg19 GTF and cDNA FASTA. Kallisto was run using default parameters with two exceptions: allowing searching for fusions (−fusion) and setting bootstrap to 100 (−b 100).

To visualize the mapped RNA-seq with IGV or UCSC genome browser, bam files were generated with Hisat 2.2.0, which was followed by making bigwig files with deeptools (v/3.0.2) (binsize 10). Reads were normalized to Reads Per Genome Coverage.

Read counts were calculated on a per transcript basis using Kallisto and the above described pseudoalignment. The R package tximport 1.10.1 was used to select the dominant transcript per gene (txOut = FALSE), which was then used for DEseq2 analysis.

#### ChIP-qPCR, ChIP-seq, and data analysis of ChIP-seq

ChIP was performed as previously described with minor modifications.^23,57^ Briefly, cells for ChIP were cultured in 15 cm plates to ~90% confluency, washed with PBS, crosslinked at room temperature with 10 mM DMP (ThermoFisher Scientific) for 30 min, and then 1% formaldehyde (ThermoFisher Scientific) for 15 min. Crosslinking reactions were quenched by addition of 125 mM glycine for 5 min at room temperature. Crosslinked cells were separated by 3 min treatment of 0.05% trypsin (Gibco), and then washed with cold PBS 3 times. In every wash, cells were centrifuged for 3 min at 1000xg at 4°C. Cells were then resuspended in sonication buffer (pH 7.9, 50 mM Hepes, 140 mM NaCl, 1 mM EDTA, 1% Triton, 0.1% Sodium deoxycholate, and 0.5% SDS) and sonicated to shear chromatin into 100-800 bp fragments using a Branson sonicator. Sonicated samples were diluted 5-fold with ChIP dilution buffer (pH 7.9, 50 mM Hepes, 140 mM NaCl, 1 mM EDTA, 1% Triton, 0.1% Sodium deoxycholate) to obtain a final concentration of 0.1% SDS. Diluted samples were centrifuged at 13,000 rpm for 3 min at 4°C. The supernatant was immunoprecipitated for 3-12 h using 3 μg antibodies and 20 μl protein A/G. The beads were washed twice with high salt wash buffer A (pH 7.9, 50 mM Hepes, 500 mM NaCl, 1 mM EDTA, 1% Triton, 0.1% Sodium deoxycholate, and 0.1% SDS), and once with wash buffer B (pH 7.9, 50 mM Hepes, 250 mM LiCl, 1 mM EDTA, 1% Triton, 0.1% Sodium deoxycholate, 0.5% NP-40). The bound chromatin fragments were eluted with elution buffer (pH 8.0, 50 Mm Tris, 10 mM EDTA, 1% SDS) for 5 min at 65°C. Eluted DNA-proteins complexes were treated with RNase A and crosslinks were reversed overnight at 65°C. Proteinase K was then added to digest proteins for 1 h at 55°C. DNA was further purified using PCR Purification Kit (28106, QIAGEN) and analyzed by PCR on a QuantStudio 7 Flex Real Time PCR System (Applied Biosystem). PCR parameters were 95°Cfor 2 min and 40 cycles of 95°C for 15s, 60°C for 15s, and 72°C for 15 s, followed by 72°C for 1 min. Primer sequences are listed in Table S2.

For ChIP-seq, sequencing library was constructed using TruSeq DNA sample Prep Kits (Illumina) and adapter dimers were removed by agarose gels electrophoresis and gel extraction (740609.250, Macherey Nagel). Sized selected and purified DNA libraries were sequenced on an Illumina Hiseq 2500 machine (Bauer core facility at Harvard University) to obtain 50 bp single-ended reads. ChIP-seq reads were quality controlled with fastqc (v0.11.5) and mapped to the human genome reference (GRCh37/hg19) using bowtie2 (v2.2.9) with default parameters. Bam files were generated with samtools 1.3.1, which was followed by making bigwig files with deeptools (v/3.0.2) (binsize 10). Reads were normalized to Reads Per Genome Coverage (RPGC) with deeptool (v/3.0.2) bamCoverage function. Peak calling was performed with MACS2 (2.1.1.20160309) with Input ChIP–seq sample as control (−p 0.05–broad, −broad-cutoff 0.05, FoldChang>2.5, Length>800 bp). To quantify ChIP-seq signal, Deeptools(v/3.0.2) multiBigwig-Summary was used to count reads that are mapped in the whole peak regions for both siCtrl and siSENP3/siUSP7 ChIP-seq samples. To analyze read density at TSS regions, we made heatmaps and metaplots of ChIP-seq samples. TSS was centered in the regions plotted and data were tabulated with the same distance relative to TSS. Matrix files were generated using computematrix function of deeptools (v/3.0.2). Based on generated matrix file, heatmaps were generated by PlotHeatmap function, and profiles were generated by plotprofile function.

### QUANTIFICATION AND STATISTICAL ANALYSIS

Statistical analyses in Figures 1, 2, and S3 were performed using two-sided Wilcoxon test. GraphPad Prism was used to make dot plots in Figures 1, 2, and S3. p values are labeled in each figure and the figure legends. For RNA-seq, upregulated genes and downregulated genes in HEK293FT cells are defined with Padj <0.05 and FoldChange >2 or <0.5. Upregulated genes and downregulated genes in human ES cells are defined with Padj <0.1 and FoldChange >1.5 or <0.67 (for SENP3 knockdown and EZH1/2 DKO) and Padj <0.05 and FoldChange >2 or <0.5 (RING1B knockdown). What N represents is indicated in Figures 1 and 3 legends. RNA-seq statistical significance for comparisons was assessed by Wilcoxon (unpaired) or Mann-Whitney (two-sided) tests. The test used and error bars are defined in each figure legend.

## Supplementary Material

ProteomicsTable

FigS1-S4_TabS1-S2

## Figures and Tables

**Table:** KEY RESOURCES TABLE

REAGENT or RESOURCE	SOURCE	IDENTIFIER
Antibodies		
Anti-RING1B	Cell Signaling Technology	Cat#5694S
Anti-CBX2	Proteintech	Cat#15579-1-AP; RRID:AB_2737362
Anti-RING1A	Cell Signaling Technology	Cat#13069S
Anti-PHC2	Elabscience	Cat#E-AB-65051
Anti-SENP3	Cell Signaling Technology	Cat#5591S
Anti-WDR18	Sigma	Cat#HPA050193; RRID:AB_2681047
Anti-USP7	Cell Signaling Technology	Cat#4833T
Anti-NOL9	Sigma	Cat#SAB4301156
Anti-BMI1	Proteintech	Cat#10832-1-AP; RRID:AB_2065392
Anti-BMI1	Proteintech	Cat#A301-694A-T
Anti-H2AK119ub1	Cell Signaling Technology	Cat#8240T
Anti-CBX8	Bethyl	Cat#A300-882A-T; RRID:AB_2632113
Anti-Flag	Sigma	Cat#F3165; RRID:AB_259529
Anti-Flag M2-Peroxidase	Sigma	Cat#A8592; RRID:AB_439702
Anti-TEX10	ThermoFisher	Cat#720257
Anti-GAPDH	Abcam	Cat#Ab181603; RRID:AB_2687666
Anti-SUMO2/3	Proteintech	Cat#11251-1-AP; RRID:AB_2198405
Anti-Ubiquitin	Proteintech	Cat#10201-2-AP; RRID:AB_671515
Anti-PCGF1	Abcam	Cat#Ab259943
Anti-EZH2	Cell Signaling Technology	Cat#5246S
Anti-EED	Millipore	Cat#17-10034; RRID:AB_10615775
Anti-SUZ12	Millipore	Cat#17-661; RRID:AB_10615481
Anti-DDB1	Proteintech	Cat#11380-1-AP; RRID:AB_2088808
Anti-RNF138	Abcam	Cat#Ab92730; RRID:AB_2238719
Anti-TRIM27	Proteintech	Cat#12205-1-AP; RRID:AB_2256660
Anti-RAD18	Proteintech	Cat#18333-1-AP; RRID:AB_2176586
Anti-P53	Proteintech	Cat#60283-2-lg
Chemicals, Peptides, and Recombinant Proteins		
FT671	MedChemExpress	HY-107985
MG132	Sigma	M8699-1MG
P5091	MedChemExpress	HY-15667
P22077	Cayman Chemical	23704
HBX19818	MedChemExpress	HY-17540
HBX41108	Cayman Chemical	23759
CX4945	MedChemExpress	HY-50855
Deposited Data		
Human embryonic kidney 293 TEX10 (siNC)	Zhou et al., 2022	GSM5343685
Human embryonic kidney 293 TEX10 (siTEX10)	Zhou et al., 2022	GSM5343687
Human embryonic kidney 293 MDN1 (siNC)	Zhou et al., 2022	GSM4239951
Human embryonic kidney 293 MDN1 (siMDN1)	Zhou et al., 2022	GSM4239949
Human embryonic kidney 293 H3K9me3	Zhou et al., 2022	GSM4239943
Human embryonic kidney 293 H3K27me3	Zhou et al., 2022	GSM4239945
Human ES H2AK119ub1	Zhou et al., 2022	GSM5343673
Human ES H3K9me3	Vallot et al., 2015	GSM1528888
Human ES H3K27me3	Vallot et al., 2015	GSM1528885
CBX2-HEK293FT_siNC_rep1	This study	GSM5907153
CBX2-HEK293FT_siNC_rep2	This study	GSM5907154
CBX2-HEK293FT_siUSP7_rep1	This study	GSM5907155
CBX2-HEK293FT_siUSP7_rep2	This study	GSM5907156
H2AK119ub1-HEK293FT_siNC_rep1	This study	GSM5907157
H2AK119ub1-HEK293FT_siNC_rep2	This study	GSM5907158
H2AK119ub1-HEK293FT_siUSP7_rep1	This study	GSM5907159
H2AK119ub1-HEK293FT_siUSP7_rep2	This study	GSM5907160
TEX10_siNC_rep1	This study	GSM5907161
TEX10_siNC_rep2	This study	GSM5907162
TEX10_siSENP3_rep1	This study	GSM5907163
TEX10_siSENP3_rep2	This study	GSM5907164
BMI1-HEK293FT_siNC_rep1	This study	GSM6896328
BMI1-HEK293FT_siNC_rep2	This study	GSM6896329
BMI1-HEK293FT_siUSP7_rep1	This study	GSM6896330
BMI1-HEK293FT_siUSP7_rep2	This study	GSM6896331
PCGF1-HEK293FT_siNC_rep1	This study	GSM6896332
PCGF1-HEK293FT_siNC_rep2	This study	GSM6896333
PCGF1-HEK293FT_siUSP7_rep1	This study	GSM6896334
PCGF1-HEK293FT_siUSP7_rep2	This study	GSM6896335
RING1B-HEK293FT_siNC_rep1	This study	GSM6896336
RING1B-HEK293FT_siNC_rep2	This study	GSM6896337
RING1B-HEK293FT_siUSP7_rep1	This study	GSM6896338
RING1B-HEK293FT_siUSP7_rep2	This study	GSM6896339
NC_rep1	This study	GSM5907165
NC_rep2	This study	GSM5907166
siCtrl_rep1	This study	GSM5907167
siCtrl_rep2	This study	GSM5907168
siSENP3_muRes_rep1	This study	GSM5907169
siSENP3_muRes_rep2	This study	GSM5907170
siSENP3_rep1	This study	GSM5907171
siSENP3_rep2	This study	GSM5907172
siSENP3_wtRes_rep1	This study	GSM5907173
siSENP3_wtRes_rep2	This study	GSM5907174
siUSP7_rep1	This study	GSM5907175
siUSP7_rep2	This study	GSM5907176
USP7in_rep1	This study	GSM5907177
USP7in_rep2	This study	GSM5907178
siNC_ES_rep1	This study	GSM5907249
siNC_ES_rep2	This study	GSM5907250
siRNF2_ES_rep1	This study	GSM5907251
siRNF2_ES_rep2	This study	GSM5907252
siSENP3_ES_rep1	This study	GSM5907253
siSENP3_ES_rep2	This study	GSM5907254
EZH_DKO_ES_rep1	This study	GSM5907255
EZH_DKO_ES_rep2	This study	GSM5907256
CBX2, CBX4, and PHC2 IP-MS	This study	PXD027966
Experimental Models: Cell Lines		
HEK293FT	Thermofisher	R70007
human ES cell	Harvard Medical School	N/A
Oligonucleotides		
EZH1 sgRNA 5’-CCGGCGACGACCAGAGCACT-3’	This study	N/A
EZH2 sgRNA 5’-TGGGGTCTTTATCCGCTCAG-3’	This study	N/A
CITRINE (CTRN)-forward 5’-CAAGGGCGAGGAGCTGTTC-3’	This study	N/A
CITRINE (CTRN)-reverse 5’-CACGCTGAACTTGTGGCCG-3’	This study	N/A
For ChIP-qPCR primers, see Table S1	This study	N/A
For RNAi sequence, see Table S2	This study	N/A
Software and Algorithms		
deepTools2	Ramírez et al., 2016	https://github.com/deeptools/deepTools
Bowtie 2	Ben Langmead	https://github.com/BenLangmead/bowtie2
MACS2	Tao Liu	https://pypi.org/project/MACS2/2.1.1.20160309/
graphpad prism 5.0	GraphPad	https://www.graphpad.com

## Highlights

- SENP3-mediated deSUMOylation regulates the association of the rixosome with PRC1
- The USP7 ubiquitin protease associates with canonical PRC1
- USP7 regulates PRC1 stability by deubiquitinating CBX2 and CBX4
- SENP3 and USP7 are required for silencing of Polycomb target genes
