# Supplementary material for: SENP3 and USP7 regulate Polycomb-rixosome interactions and silencing functions
Source: Cell Rep. Author manuscript; Available in PMC 2024 Apr 25. (PMC10777863; doi:10.1016/j.celrep.2023.112339)
Supplement: FigS1-S4_TabS1-S2 [file NIHMS1954671-supplement-FigS1-S4_TabS1-S2.pdf]

Cell Reports, Volume *42*

Supplemental information

SENP3 and USP7 regulate Polycomb-rixosome interactions and silencing functions

Haining Zhou, Wenzhi Feng, Juntao Yu, Tiasha A. Shafiq, Joao A. Paulo, Jiuchun Zhang, Zhenhua Luo, Steven P. Gygi, and Danesh Moazed

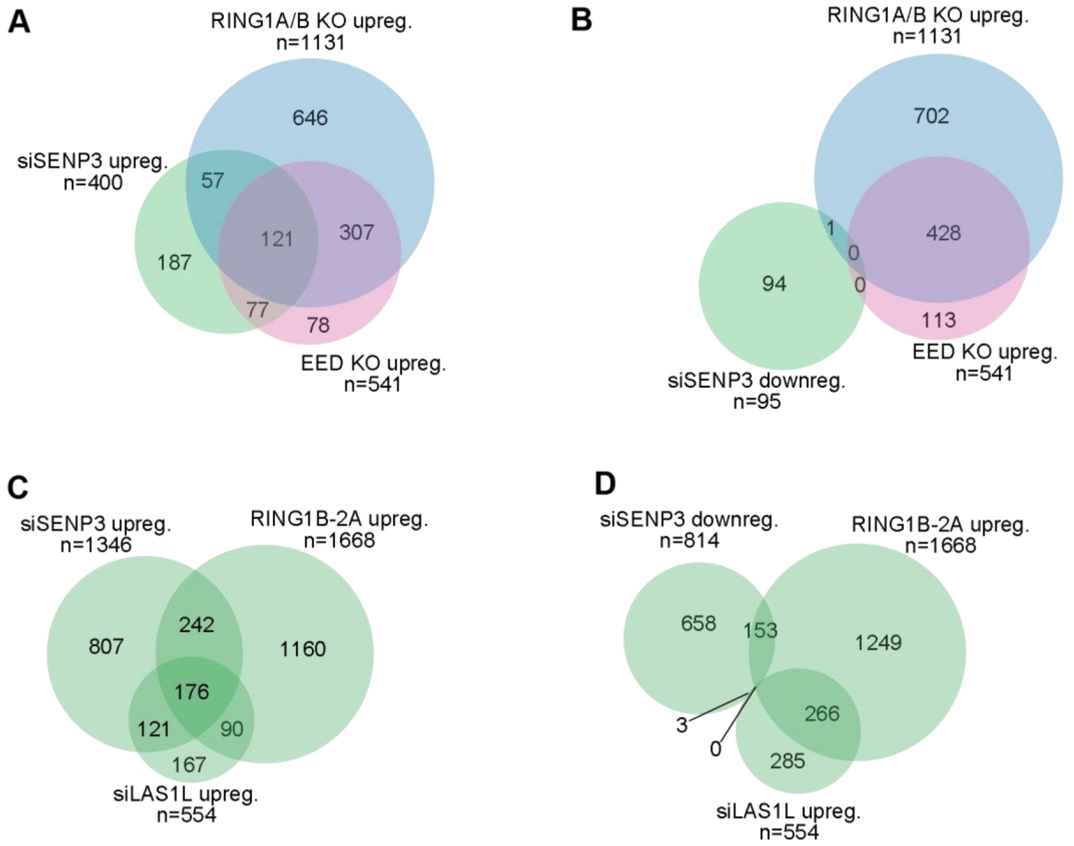

Figure S1. Depletion of SENP3 and LAS1L results in upregulation of overlapping genes. Related to Figure 1.

A, Venn diagrams showing overlap among upregulated genes in SENP3 KD with upregulated genes in *EED* KO and *RING1A/B* DKO cells with a fold change cutoff of >5 in RNA-seq experiments. Hypergeometric probability *P* values: siSENP3 upreg vs. *RING1A/B* DKO, 4e-374; siSENP3 upreg vs. *EED* KO, 2e-354.

B, Venn diagrams showing overlap among upregulated genes in *SENP3 KD* with downregulated genes in *EED* KO and *RING1A/B* DKO cells with a fold change cutoff of >5 in RNA-seq experiments. Hypergeometric probability *P* values: siSENP3 upreg vs. *RING1A/B* DKO, 1.5e-7; siSENP3 upreg vs. *EED* KO, 1.8e-8.

C, Venn diagrams of RNA-seq results showing overlap among upregulated genes in siSENP3, siLAS1L, and RING1B-2A mutant cells.

D, Venn diagrams of RNA-seq results showing overlap of downregulated genes in siSENP3 with upregulated genes in siLAS1L and RING1B-2A mutant cells.

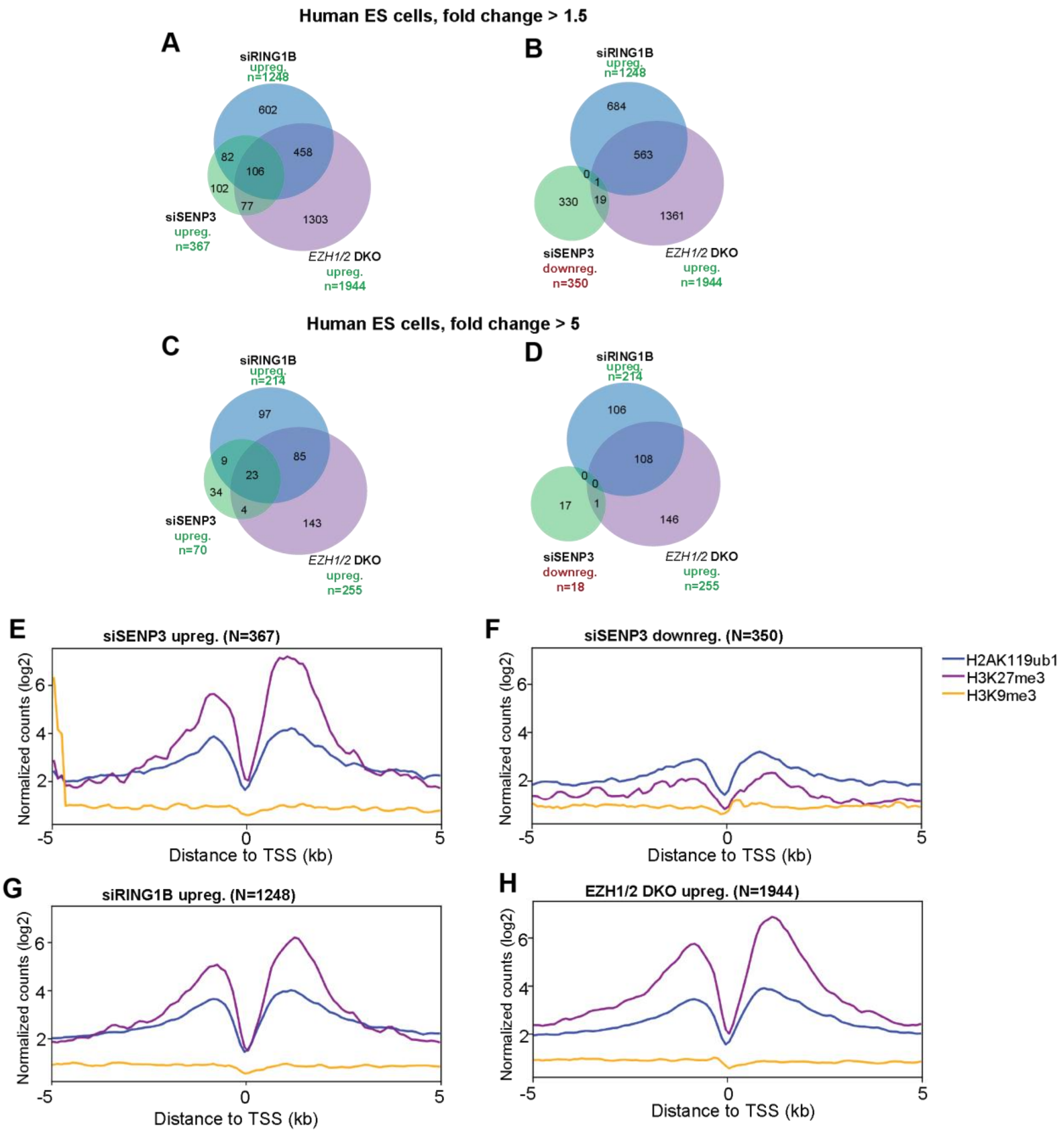

Figure S2. SENP3 is required for Polycomb-target gene repression in human ES cells. Related to Figure 2.

A, Venn diagrams of RNA-seq results showing overlap among upregulated genes in siSENP3 with upregulated genes in *EZH1/2* DKO and siRING1B cells in human ES cells. Hypergeometric

probability *P* values: siSENP3 upreg vs. *EZH1/2* DKO, 1.2e-98; siSENP3 upreg vs. *RING1B* KD, 3.6e-140.

B, Venn diagrams of RNA-seq results showing overlap among downregulated genes in siSENP3 with upregulated genes in *EZH1/2* DKO and siRING1B cells in human ES cells. Hypergeometric probability *P* values: siSENP3 downreg vs. *EZH1/2* DKO, 0.04; siSENP3 downreg vs. *RING1B* KD, 7.3e-8.

C, Same as in **A** but showing >5 fold changes in gene expression.

D, Same as in **B** but showing >5 fold changes in gene expression.

E-F, Average distribution of the indicated ChIP-seq (two biological replicates) reads (log2) for genes upregulated (**C**) and downregulated genes (**D**) in siSENP3 RNA-seq experiments from human ES cells. Enrichment levels were normalized with Reads Per Genome Coverage. Read counts per gene were summed in 50-nt bins.

G-H, Average distribution of the indicated ChIP-seq (two biological replicates) reads (log2) for genes upregulated in siRING1B (**E**) and *EZH1/2* DKO (**F**) RNA-seq experiments from human ES cells. Enrichment levels were normalized with Reads Per Genome Coverage. Read counts per gene were summed in 50-nt bins.

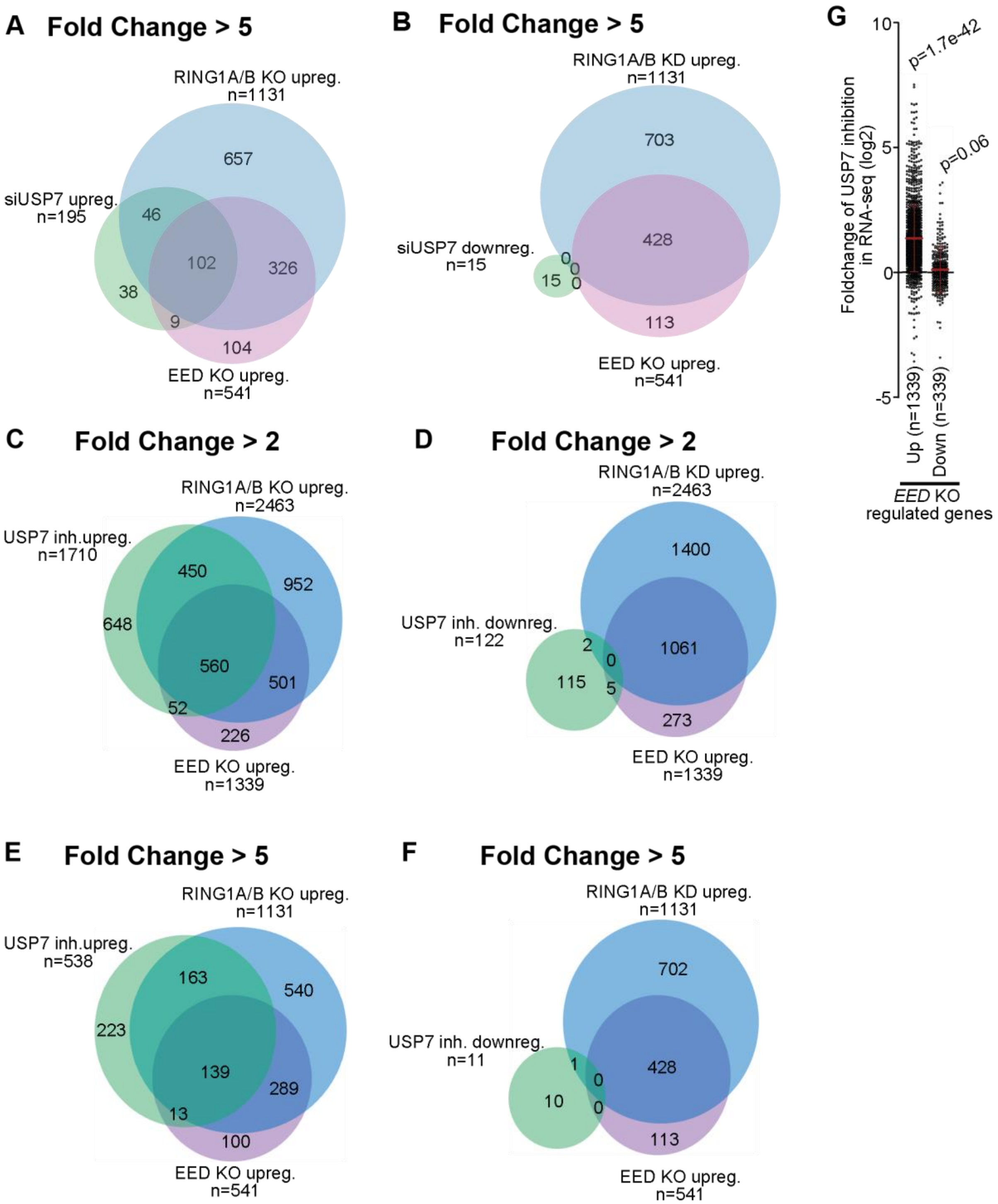

Figure S3. Inhibition of Deubiquitylation activity of USP7 derepresses Polycomb-target genes. Related to Figure 3.

A, Venn diagrams showing overlap among upregulated genes in siUSP7 with upregulated genes in *EED* KO and *RING1A/B* DKO cells with a fold change cutoff of >5 in RNA-seq experiments. Hypergeometric probability *P* values: siUSP7 upreg vs. *RING1A/B* DKO, 2.8e-517; siUSP7 upreg vs. *EED* KO, 9.6e-393.

B, Venn diagrams showing overlap among downregulated genes in siUSP7 with upregulated genes in *EED* KO and *RING1A/B* DKO cells with a fold change cutoff of >5 in RNA-seq experiments. Hypergeometric probability *P* values: siUSP7 downreg vs. *RING1A/B* DKO, 0.2; siSENP3 downreg vs. *EED* KO, 0.4.

C, Venn diagrams showing overlap among upregulated genes in USP7 inhibition with upregulated genes in *EED* KO and *RING1A/B* DKO cells in RNA-seq experiments. Hypergeometric probability *P* values: USP7 inhibition upreg vs. *RING1A/B* DKO, 6e-601; USP7 inhibition upreg vs. *EED* KO, 9.9e-362.

D, Venn diagrams showing overlap among downregulated genes in USP7 inhibition with upregulated genes in *EED* KO and *RING1A/B* DKO cells in RNA-seq experiments. Hypergeometric probability *P* values: USP7 inhibition downreg vs. *RING1A/B* DKO, 1.3e-4; USP7 inhibition downreg vs. *EED* KO, 0.3.

E, Same as in **C** but showing >5 fold changes in gene expression.

F, Same as in **D** but showing >5 fold changes in gene expression.

G, Dot plots showing RNA-seq changes of USP7 inhibition (1 uM FT671 for 3 days) compared to control in the *EED* KO-upregulated and -downregulated sets of genes in RNA-seq experiments in HEK293FT cells. Data are presented as mean values +/- SEM. *P* value is from the two-tailed Wilcoxon test.

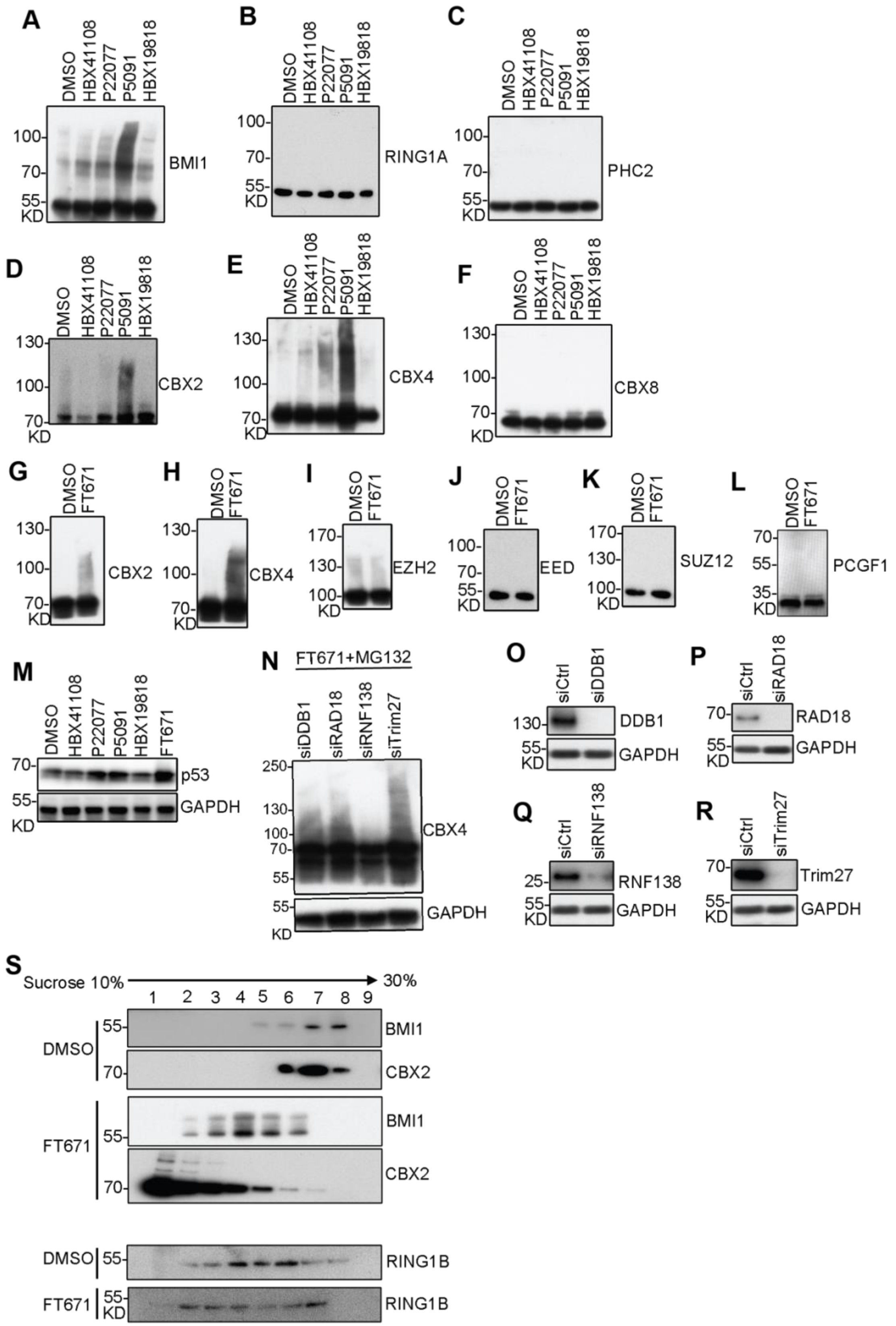
A
DMSO HBX41108 P22077 P5091 HBX19818
100- 70- 55- KD
BMI1
B
DMSO HBX41108 P22077 P5091 HBX19818
100- 70- 55- KD
RING1A
C
DMSO HBX41108 P22077 P5091 HBX19818
100- 70- 55- KD
PHC2
D
DMSO HBX41108 P22077 P5091 HBX19818
130- 100- 70- KD
CBX2
E
DMSO HBX41108 P22077 P5091 HBX19818
130- 100- 70- KD
CBX4
F
DMSO HBX41108 P22077 P5091 HBX19818
130- 100- 70- KD
CBX8
G
DMSO FT671
130- 100- 70- KD
CBX2
H
DMSO FT671
130- 100- 70- KD
CBX4
I
DMSO FT671
170- 130- 100- KD
EZH2
J
DMSO FT671
100- 70- 55- KD
EED
K
DMSO FT671
170- 130- 100- KD
SUZ12
L
DMSO FT671
70- 55- 35- KD
PCGF1
M
DMSO HBX41108 P22077 P5091 HBX19818 FT671
70- p53
55- GAPDH
KD
N
FT671+MG132
siDDB1 siRAD18 siRNF138 siTrim27
250- 130- 100- 70- 55-
CBX4
55- GAPDH
KD
O
siCtrl siDDB1
130- DDB1
55- GAPDH
KD
P
siCtrl siRAD18
70- RAD18
55- GAPDH
KD
Q
siCtrl siRNF138
25- RNF138
55- GAPDH
KD
R
siCtrl siTrim27
70- Trim27
55- GAPDH
KD
S
Sucrose 10% → 30%
1 2 3 4 5 6 7 8 9
DMSO
55- BMI1
70- CBX2
FT671
55- BMI1
70- CBX2
DMSO 55- RING1B
FT671 55- RING1B
KD

Figure S4. USP7 deubiquitinates PRC1 subunits of CBX2, CBX4, and BMI1. Related to Figure 4.

A-F, Immunoblots showing the migration of cPRC1 subunits in whole cell lysates from HEK293FT cells treated with 20 uM of the indicated small molecules for USP7 inhibition and 5 uM MG132 for 1 day.

G-L, Immunoblots showing the migration of the indicated PRC subunits in whole cell lysates from HEK293FT cells treated with 1 uM FT671 USP7 inhibitor and 5 uM MG132 for 1 day.

M, Immunoblot showing P53 protein levels in whole cell lysates from HEK293FT cell treated with 20 uM indicated small molecules for USP7 inhibition.

N, Immunoblot showing Flag-CBX4 levels in HEK293FT cells treated with the indicated siRNA (3 days), 5 uM MG132, and 1 uM FT671 for 1 day. GAPDH served as input control.

O-R, Immunoblots showing expression levels of the indicated proteins in HEK293FT cells treated with siCtrl or the indicated siRNA (3 days). GAPDH served as loading control.

S, Migration of PRC1 subunits BMI, CBX2, and RING1B in sucrose gradients. DMSO or FT671 (1 uM) treated cells were subjected to 10–30% sucrose gradient sedimentation. Fractions were collected and adsorbed to Strataclean beads and analyzed by immunoblotting with the indicated antibodies. Top, Flag-CBX2 immunopurified proteins; bottom, Flag-RING1B immunopurification.

Table S1. SiRNA sequences used in this study.

Sequence	Source	Identifier
SENP3	**This study**	5’-GGGAGUCCCUUCCCAAGAA-3’
USP7	**This study**	5’-ACCCUUGGACAAUAUUCCU-3’
NSMCE2	**This study**	5’-GGATCTTGTGGAAAGTCAGACTGAA-3’
PIAS1	**This study**	5’-CAGCAAATCAGTAGTTCCATGGATA-3’
PIAS2	**This study**	5’-CCTGTCCATCCTGATGTGCAGTTAA-3’
PIAS3	**This study**	5’-CAGCGGTTTGAGGAAGCGCACTTTA-3’
RANBP2	**This study**	5’-CACCTCCTGTGTGGTTACAAGTTAA-3’
CBX4	**This study**	5’-CGGCCTCAGAGTTCTAGTATTATAT-3’
TRIM27	**This study**	5’-GAGCTAGACTTGGCCATCTACAATA-3’
RNF138	**This study**	5’-AACCATTCTGGGATTTGCAAAGTTT-3’
DDB1	**This study**	5’-CAGTAATGAACAAGGCTCCTATGTA-3’
RAD18	**This study**	5’-GAGTCTCATTATGGCTGAGAGTTTA-3’
CSNK2A1	**This study**	5’-GUCCGAGUUGCUUCCCGA-3’
CSNK2A2	**This study**	5’-UACACGAACAUUGUACUCC-3’
CSNK2B	**This study**	5’-CTCAGAGGAGGTGTCCTGG-3’
Control	**This study**	5’-UUCUCCGAACGUGUCACGU-3’

Table S2. ChIP-qPCR primers used in this study.

Sequence	Source	Identifier
P1-forward	**This study**	5'-GGAATTCCAGATGGTGCGCT-3'
P1-reverse	**This study**	5'-GGCCAGAGCAGATACGTAGG-3'
P2-forward	**This study**	5'-ACGTATGTCGAGGTAGGCGT-3'
P2-reverse	**This study**	5'-CTAGGCACCGGTTCAATTGC-3'
P3-forward	**This study**	5'-CGCCTTTTT CCCGAGGGTG-3'
P3-reverse	**This study**	5'-GTGTTCTGG CGGCAAACCC-3'
P4-forward	**This study**	5'-TGCATAAACGTTGTCGCCATT-3'
P4-reverse	**This study**	5'-AAGTGCGCCCTTCGAGTAAG-3'
